# Characterization and Biotechnological Potential Analysis of a New Exopolysaccharide from the Arctic Marine Bacterium *Polaribacter* sp. SM1127

**DOI:** 10.1038/srep18435

**Published:** 2015-12-21

**Authors:** Mei-Ling Sun, Fang Zhao, Mei Shi, Xi-Ying Zhang, Bai-Cheng Zhou, Yu-Zhong Zhang, Xiu-Lan Chen

**Affiliations:** 1 State Key Laboratory of Microbial Technology, Jinan 250100, China; 2 Marine Biotechnology Research Center, Shandong University, Jinan 250100, China

## Abstract

Although many kinds of exopolysaccharides (EPSs) from microorganisms have been used in industry, the exploration and utilization of EPSs from polar microorganisms is still rather rare. In this study, a flavobacterial strain, SM1127, from the Arctic brown alga *Laminaria*, was screened for its high EPS production (2.11 g/l) and was identified as belonging to the genus *Polaribacter*. The EPS secreted by strain SM1127 has a molecular mass of 220 kDa, and it mainly comprises *N*-acetyl glucosamine, mannose and glucuronic acid residues bound by heterogeneous linkages. Rheological studies on the aqueous EPS showed that it had a high viscosity and good shear-thinning property. Moreover, the EPS showed a high tolerance to high salinity and a wide pH range. The EPS also had good antioxidant activity. Particularly, its moisture-retention ability was superior to that of any other reported EPS or functional ingredient generally used in cosmetics. The EPS also showed a protective effect on human dermal fibroblasts at low temperature (4 °C). Safety assessment indicated that the EPS is safe for oral administration and external use. These results indicate the promising potential of the EPS from strain SM1127 in the food, cosmetic, pharmaceutical and biomedical fields.

In marine environments, most microbial cells are surrounded by a layer of extracellular carbohydrate polymers, which usually are exopolysaccharides (EPSs)^1^. EPSs, with linear or branched structures, are composed of repeating units of different monosaccharides or their derivatives^2^. They help microbes survive in diverse marine environments by influencing the physicochemical environment near the microbial cell^3^. Some extreme marine environments, such as deep-sea hydrothermal vents, shallow submarine thermal springs and polar marine habitats, have been regarded as new sources for the exploration of EPS-producing bacteria^3^. Studying marine bacterial EPSs will advance our understanding of marine bacteria and offer more opportunities to find new EPS resources for biotechnological and industrial application.

Natural EPSs, such as xanthan gum and hyaluronic acid (HA), have been widely applied in food, medicine, cosmetics and nutraceuticals, etc^4^,^5^. Although a number of bacteria from various marine environments have been found to secrete EPSs, only a few EPSs from marine bacteria have been characterized and developed for their application potential^3^. For example, the EPS secreted by *Alteromonas* sp. strain 1644, isolated near a hydrothermal vent of the East Pacific Rise, shows great affinity for some divalent ions, which can be useful in wastewater treatment^6^,^7^. The EPS extracted from *Zunongwangia profunda* SM-A87 from deep-sea sediment also has promising potential in wastewater treatment due to its metal-binding capacity for Cu(II) and Cd(II)^8^. EPSs from marine bacteria are also reported to have potential applications in the pharmaceutical and medical fields^9^,^10^,^11^,^12^. Mauran, a highly polyanionic sulfated EPS produced by the halophilic bacterium *Halomonas maura,* has antioxidant, antihemolytic and antithrombogenic activities^13^. The EPS produced by a marine *Vibrio* strain exhibits antitumor, antiviral, and immunostimulant activities, and it has the potential to be developed into a therapeutic agent against cancer or other diseases^14^. An oversulfated EPS derived from a polysaccharide secreted by *Alteromonas infernus* isolated from the vicinity of a hydrothermal vent can enhance the proliferation of human umbilical vein endothelial cells, and it is potentially useful for accelerating vascular wound healing^15^.

Owing to the unique environmental conditions of polar regions, the EPSs secreted by microbes from polar habitats often have novel structures and properties^16^. For example, the EPS secreted by the Arctic sea ice bacterium *Pseudoalteromonas* sp. SM20310 is composed of a predominant repeating unit of highly complicated α-mannan and can improve the tolerance of *Escherichia coli* and strain SM20310 to freeze-thaw cycles^17^. The EPSs produced by the Antarctic bacterium *Pseudoalteromonas arctica* KOPRI 21653 and the Antarctic fungus *Phoma herbarum* CCFEE 5080 are also reported to have cryoprotective effects on the cells of these microorganisms^18^,^19^.

In the present study, strain SM1127 with high EPS production was isolated from the Arctic brown alga *Laminaria* and was identified as *Polaribacter*. The EPS produced by strain SM1127 was purified, and its glycosyl composition and linkage were characterized. Furthermore, the rheological properties, moisture-absorption and retention abilities, antioxidant activity, low-temperature protective effect on human dermal fibroblasts and safety in use of SM1127 EPS were investigated to explore its potential in biotechnology and industry.

## Results

### Screening and identification of strain SM1127

**Figure 1:**
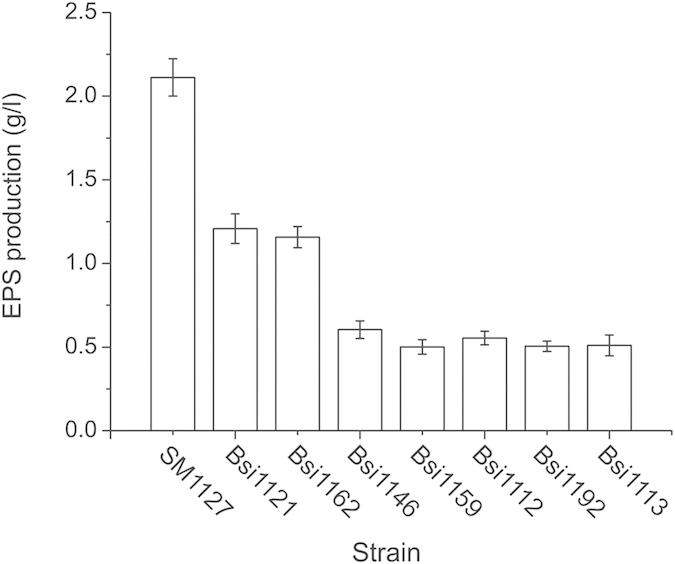
EPS production of the 8 screened strains. EPS production was determined by using the phenol-sulfuric acid method with glucose as a standard. The graph shows data from triplicate experiments (mean ± S.D.).

Using Congo red agar plates, 8 isolates that secreted mucous EPS were screened from 152 Arctic isolates that were previously isolated from the Arctic brown alga *Laminaria*. The EPS production of these isolates in liquid marine medium was then measured (Fig. 1). Isolate SM1127 had the highest EPS production (2.11 g/l) among the 8 isolates under our culture conditions, and it was therefore chosen for further study.

Strain SM1127 shared the highest 16S rRNA gene sequence similarity to *Polaribacter sejongensis* KOPRI 21160^T^ (99.1%), *Polaribacter butkevichii* KMM 3938^T^ (98.7%) and *Polaribacter irgensii* 23-P^T^ (97.4%). In the neighbor-joining phylogenetic tree (see Supplementary Fig. S1 online), strain SM1127 was grouped within the genus *Polaribacter*. Thus, strain SM1127 was phylogenetically affiliated with the genus *Polaribacter* and was named *Polaribacter* sp. SM1127.

### Purification and structural characterization of the EPS from strain SM1127

EPS was isolated from the SM1127 culture by ethanol precipitation, and proteins were removed from the EPS by protease hydrolysis. The obtained crude EPS was further purified by anion-exchange chromatography and gel-filtration chromatography. Two EPS peaks were eluted from the DEAE-Sepharose Fast Flow anion-exchange chromatographic column (see Supplementary Fig. S2 online). The first fraction was too scarce to collect, and the second large fraction was collected for further purification by a Sepharose 4B gel-filtration chromatographic column (see Supplementary Fig. S3 online). The single fraction eluted from the gel-filtration chromatographic column was collected and analyzed by a UV-Vis absorption spectrum and a Shimadzu analytical HPLC system. There was no obvious absorption at 260 nm or 280 nm in the UV-Vis absorption spectrum, indicating that there was little nucleic acid or protein in the purified EPS. Only one symmetrical acute peak was detected on the Shimadzu analytical HPLC system (see Supplementary Fig. S4 online), indicating that the EPS sample consisted of a single homogeneous component and could be used for structural characterization analysis. Size-exclusion chromatography indicated that the molecular mass of the purified EPS is approximately 220 kDa.

Glycosyl composition analysis was performed by GC/MS (see Supplementary Fig. S5 online). The results showed that it consists mostly of *N*-acetyl glucosamine, mannose, glucuronic acid, with moderate amounts of galactose and fucose and minor amounts of glucose and rhamnose. The percentages of the monosaccharide types are shown in Table 1. Glycosyl linkage analysis showed that the EPS is mainly composed of 4-linked glucuronopyranose, 2-linked galactopyranose, terminally linked galactopyranose, 4-linked glucopyranose, terminally linked fucopyranose and 2, 3-linked mannopyranose, with minor peaks for other linkages (Table 2; see Supplementary Fig. S6 online). The existence of 2, 3-linked mannopyranosyl residue indicates that the EPS molecule is hyper-branched.

### Properties of the EPS from strain SM1127

**Figure 2:**
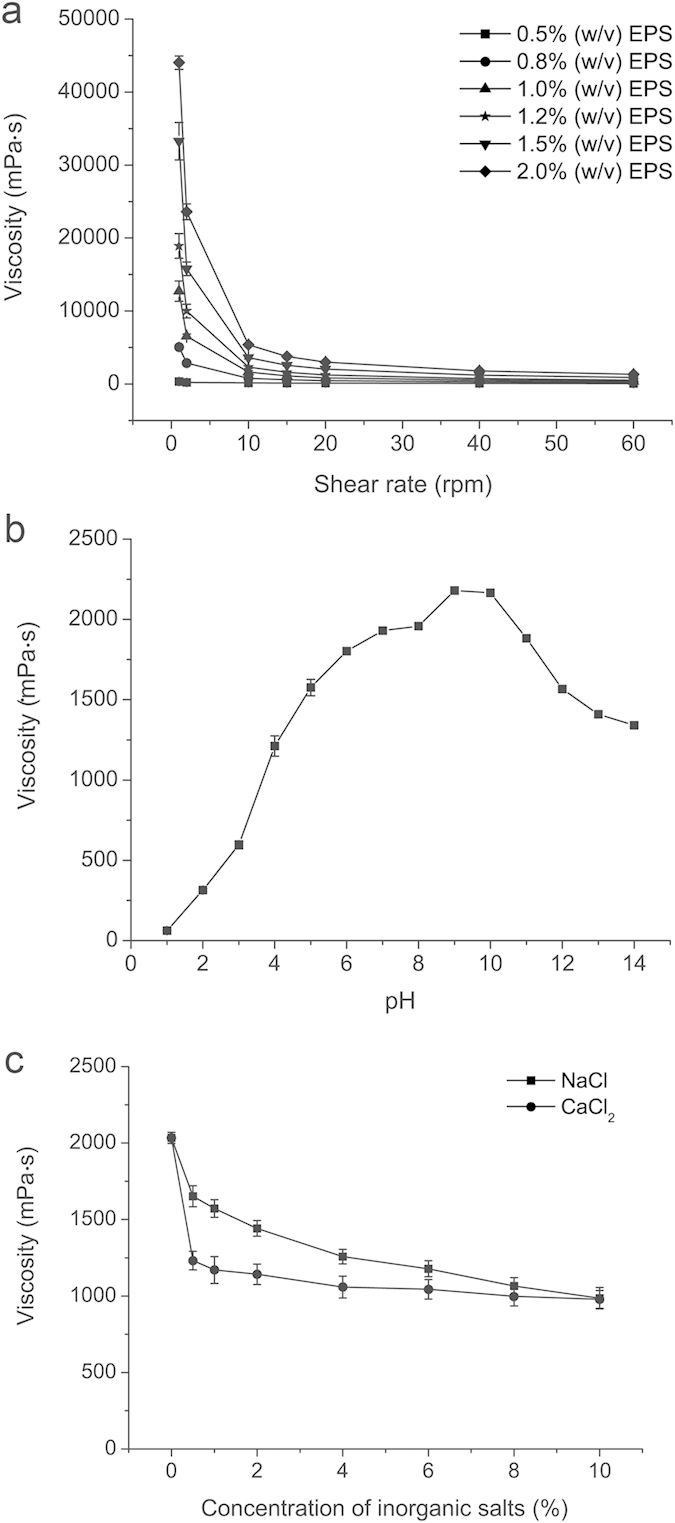
Effect of shear rate, pH and inorganic salts on the viscosity of the crude EPS solution of strain SM1127. Viscosity was measured by using a Brookfield viscometer with spindle S16. (**a**) The effect of different shear rates on the viscosity of the EPS solutions at different concentrations (0.5%, 0.8%, 1.0%, 1.2%, 1.5% and 2.0% [w/v]). (**b**) The effect of pH (1–14) on the viscosity of 1.5% (w/v) EPS solution (25 °C). (**c**) The effect of different concentrations of NaCl and CaCl_2_ (0–10%) on the viscosity of 1.5% (w/v) EPS solution (25 °C). The graph shows data from triplicate experiments (mean ± S.D.).

Figure 2a shows the relationship between different concentrations of the crude EPS aqueous solution and different shear rates. At the same shear rate, the viscosity of the EPS solution increased with concentration, indicating the EPS shows typical non-Newtonian behavior. In the meantime, the viscosity of the EPS solution decreased as the shear rate went up, showing its strong shear-thinning behavior. The apparent viscosity of the EPS solution varied slightly upon changes in pH from 5 to 12 (Fig. 2b) and salt (NaCl or CaCl_2_) concentration from 1% to 10% (Fig. 2c), indicating that the EPS had good stability under the conditions of a wide pH range and high concentration inorganic salts.

### Moisture-absorption and retention ability and antioxidant activity of the EPS from strain SM1127

**Figure 3:**
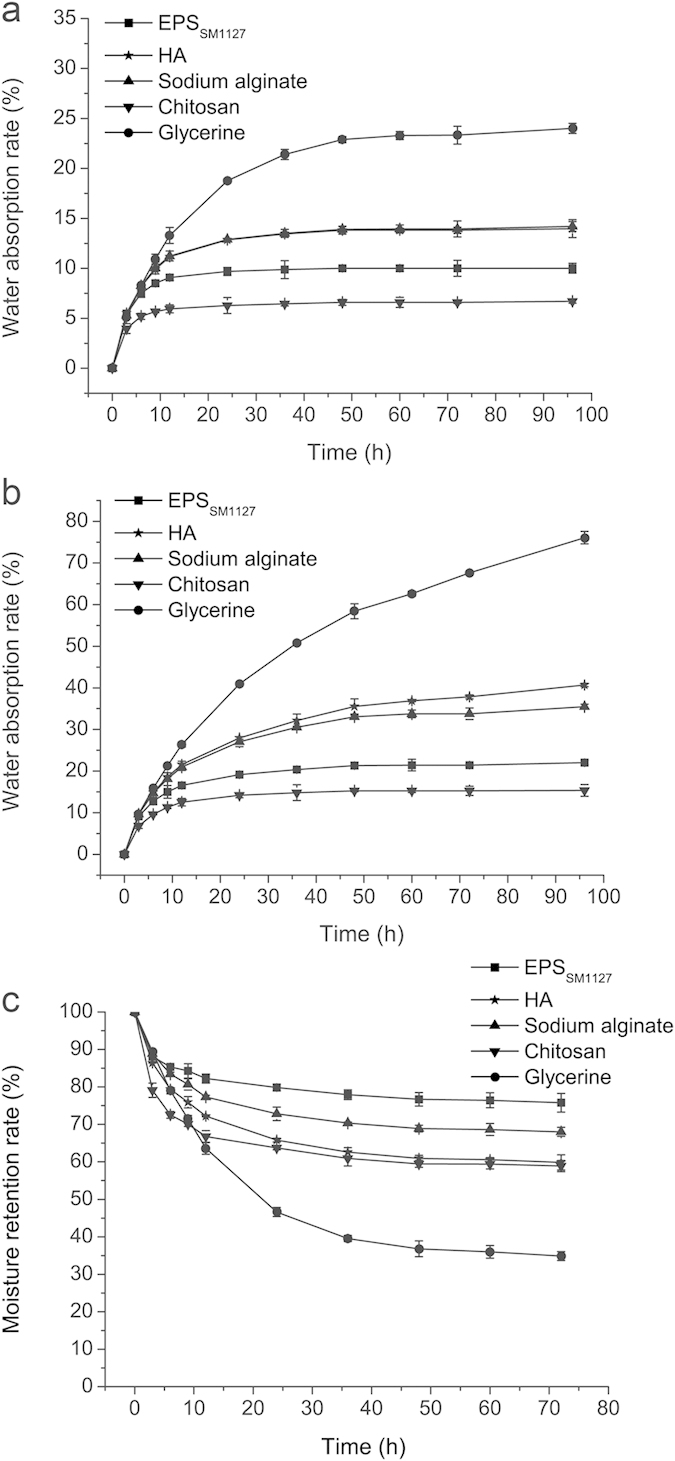
Moisture-absorption and retention abilities of SM1127 EPS and control samples. (**a**) Moisture-absorption ability in a saturated K_2_CO_3_ chamber (43% RH) at 25 °C. (**b**) Moisture-absorption ability in a saturated (NH_4_)_2_SO_4_ chamber (81% RH) at 25 °C. (**c**) Moisture-retention ability in a silica gel chamber at 25 °C. The ability was examined gravimetrically. The graph shows data from triplicate experiments (mean ± S.D.).

HA, chitosan, sodium alginate and glycerol are frequently used as humectant agents in industry^20^, and they were used as positive controls in the analysis of the moisture-absorption and retention abilities of the EPS from strain SM1127. The moisture-absorption ability (*R*_*a*_) of SM1127 EPS and the four control samples was measured at 43% RH or 81% RH. As shown in Fig. 3a, the *R*_*a*_ of all the samples at 43% RH rose gradually in the first 24 h, and while the *R*_*a*_ of glycerol continued to rise after 24 h, those of the other samples flattened out. After 72 h, the ranking for the *R*_*a*_ of all samples was as follows: glycerol >HA >sodium alginate >SM1127 EPS >chitosan. The tendency and ranking of the *R*_*a*_ of the samples at 81% RH were similar to those at 43% RH (Fig. 3b). These results showed that the *R*_*a*_ of SM1127 EPS was lower than that of most commercial agents. However, the moisture-retention ability (*R*_*h*_) of SM1127 EPS was higher than that of any tested commercial agent, reaching 75.79 ± 2.5% after the EPS was dehydrated for 72 h in a silica gel chamber (Fig. 3c).

**Figure 4:**
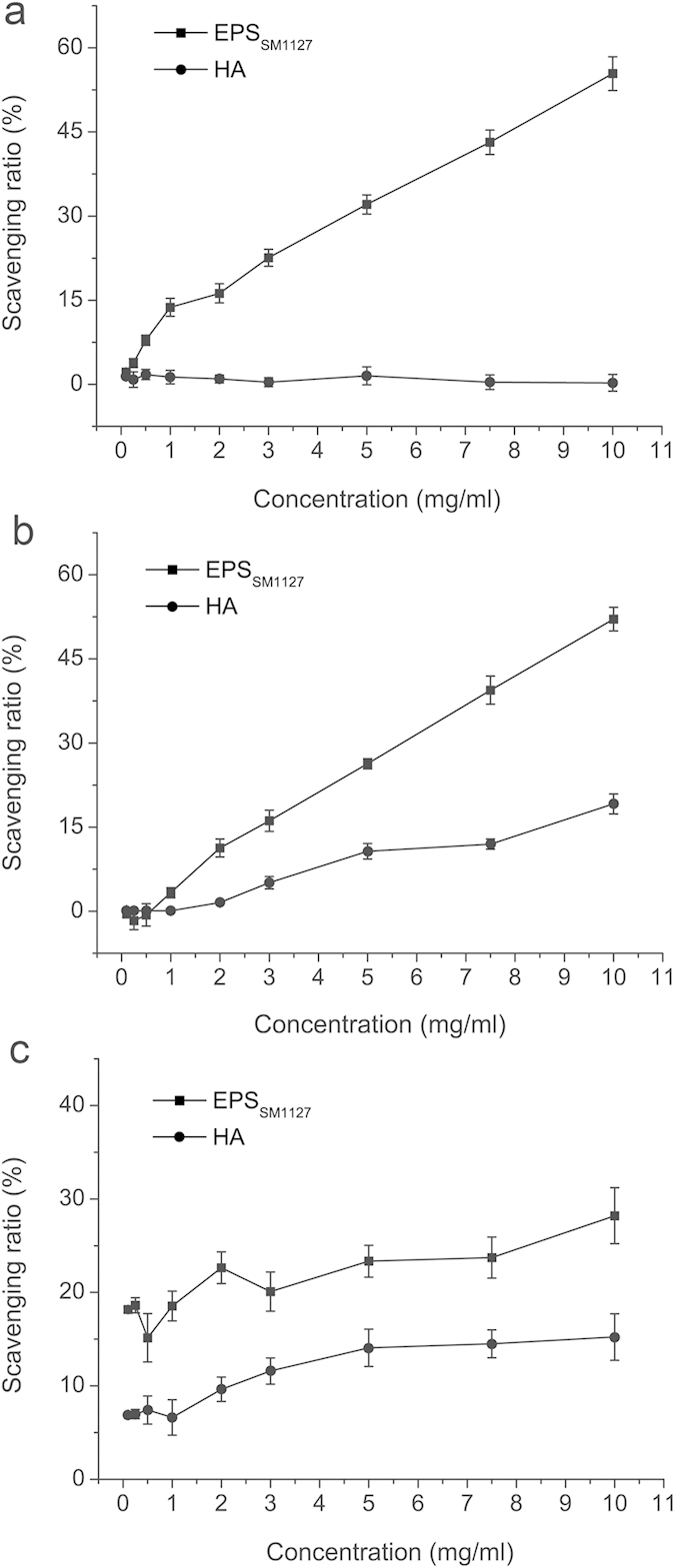
Free radical-scavenging capacities of SM1127 EPS and hyaluronic acid (HA). (**a**) The DPPH• scavenging capacity (**b**) The •OH scavenging capacity. (**c**) The O_2_^–^• scavenging capacity. The graph shows data from triplicate experiments (mean ± S.D.).

The antioxidant activity of SM1127 EPS was assessed according to its free-radical-scavenging activity. The scavenging ratios for DPPH•, •OH and O_2_^–^• of the EPS at a concentration of 10.0 mg/ml were 55.40 ± 3% (Fig. 4a), 52.1 ± 2.1% (Fig. 4b), and 28.2 ± 3% (Fig. 4c), respectively, substantially higher than those of HA, a common annexing agent in cosmetics capable of scavenging radicals^21^. These data indicate that SM1127 EPS has good antioxidant activity.

### Low-temperature protective effect of the EPS from strain SM1127 on human dermal fibroblasts

**Figure 5:**
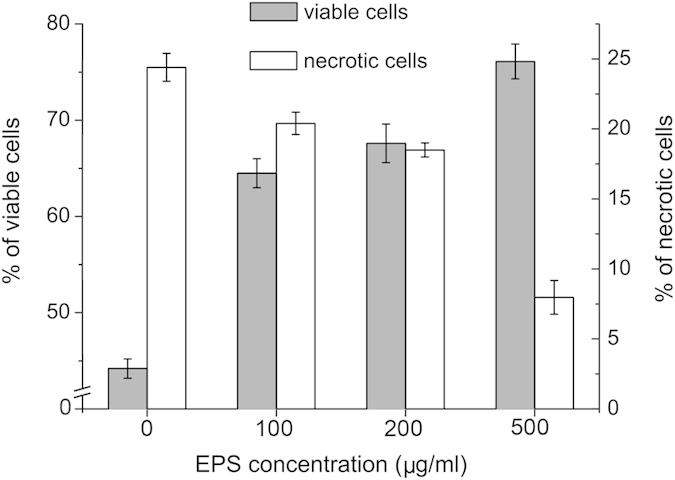
Percentage of viable and necrotic human dermal fibroblasts after a 20-h incubation in different concentrations of SM1127 EPS at 4 °C. The graph shows data from triplicate experiments (mean ± S.D.).

To investigate whether the EPS from strain SM1127 has a low-temperature protective effect on human skin, we studied its effect on the viability of human dermal fibroblasts at 4 °C. After human dermal fibroblasts were inoculated at 4 °C for 20 h with different concentrations of the EPS, the viable and necrotic cells in the samples were detected by flow cytometry (see Supplementary Fig. S7 online), and their percentages were calculated. As shown in Fig. 5, as the concentration of the EPS increased, the percentage of viable cells significantly increased, and the percentage of necrotic cells decreased substantially. When the EPS concentration in the medium reached 500 μg/ml, the percentage of viable cells was 76.1%, 31.9% higher than that in the medium without EPS (44.2%), and the percentage of necrotic cells was 7.97%, 16.4% lower than that in the medium without EPS (24.4%). This result suggests that SM1127 EPS may have a good low-temperature protective effect on human skin.

### Safety assessment of the EPS from strain SM1127

**Figure 6:**
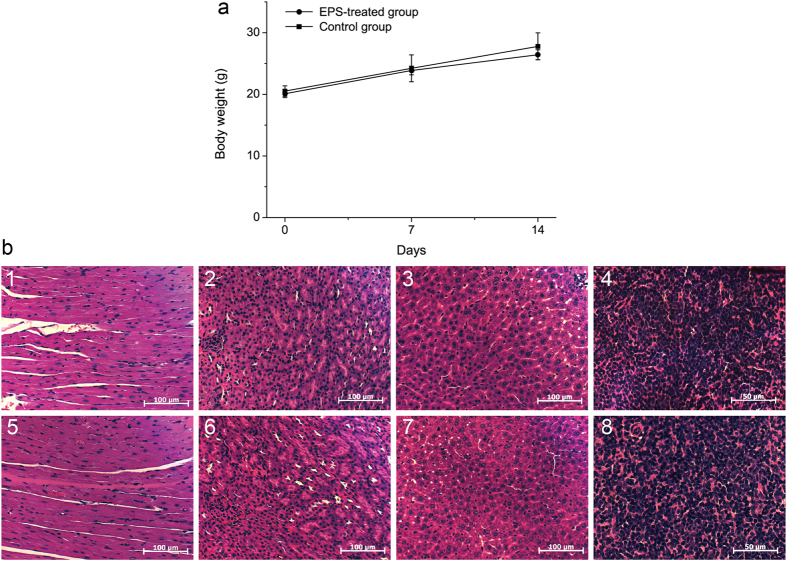
Effect of SM1127 EPS on the body weight (a) and organs (b) of KM mice in a 14-day feeding test. In graph (**a**) data are shown as the mean weight of five mice in each group (mean ± SEM). In graph (**b**) 1 and 5 are heart sections (200×); 2 and 6 are kidney sections (200×); 3 and 7 are liver sections (200×); 4 and 8 are spleen sections (400×). 1, 2, 3 and 4 were H_2_O-treated and 5, 6, 7 and 8 were EPS-treated. The sections were stained with hematoxylin-eosin (HE) and observed by light microscopy (Axio Imager. A2, Zeiss, Germany). The photographs were taken by Axio Vision 4.8.2 software.

To assess the safety of the EPS from strain SM1127, we performed an acute toxicity test on mice and a skin irritation test on rabbits. In the acute toxicity test, a dose of 5000 mg/kg of the EPS was orally administered. In the 14-day test period, no mice died in either the EPS-treated or the control group, and no clinical signs, such as hair loss, wound formation, anorexia, dull eyes, insensitivity, breathing difficulty or any other toxicological effects were observed (data not shown). The mean body weight of mice in both the treated and the control groups increased gradually and did not show a substantial difference at the end of the test (Fig. 6a). Moreover, there was no substantial increase in the incidence of histopathological changes (such as cell degeneration or accumulation) in the heart, kidney, liver or spleen of the treated mice compared with the control (Fig. 6b). Thus, it could be concluded that the LD_50_ of SM1127 EPS is higher than 5000 mg/kg.

In the skin irritation test, primary cutaneous irritation and cumulative cutaneous irritation were tested with the EPS at 125 mg/ml. In the primary cutaneous irritation test, no erythema, edema, rough or thinning skin were observed in either the control or the treated group after 1, 24, 48 or 72 h. Thus, the total score was zero, and the PII of the EPS was equal to zero according to Supplementary Table S1 online. Moreover, there was also no erythema or edema observed throughout the cumulative cutaneous irritation test. The total score of the 14-day test was zero, and the CII of the EPS was zero according to Supplementary Table S1 online. Therefore, the PII and CII were evaluated as no irritation according to Supplementary Table S2 online, which indicates that the EPS is nonirritating to skin.

## Discussion

The genus *Polaribacter*, a member of the family *Flavobacteriaceae*, was originally proposed by Gosink *et al.*^22^ to accommodate aerobic, psychrophilic and psychrotrophic bacteria. To date, it comprises thirteen species with validly published names^23^. Bacteria affiliated with the genus *Polaribacter* have been found in different marine environments, especially in the Antarctic and Arctic regions^24^,^25^. In this study, we screened an EPS-secreting strain of *Polaribacter* sp. SM1127.

There has been only one report on EPS secreted by *Polaribacter* before. Nichols *et al.* reported the glycosyl composition of the EPS secreted by an Antarctic marine bacterium within the genus *Polaribacter*^26^. In this study, the glycosyl composition and linkage of the EPS from *Polaribacter* sp. SM1127 were analyzed, revealing them to be different from those of the EPSs secreted by other marine bacteria^26^,^27^,^28^. Property analysis showed that a solution of the EPS from strain SM1127 has non-Newtonian pseudoplastic characteristics and high viscosity, making it have potential advantages in food processing as a thickener as well as in industrial operations as a mixing agent^29^. Moreover, the SM1127 EPS solution has good pH stability and salt tolerance, which would help to broaden its applications.

Moisture-absorbing and retaining materials have extensive applications in food, cosmetic, pharmaceutical and other industries^30^. Among them, glycerin is the most conventional material, and it has good moisture-absorption ability. However, the low moisture-retention rate limits its application. HA has a high moisture-retention rate and is a significant functional ingredient in modern cosmetics^30^. Our result showed that the moisture-retention ability of SM1127 EPS is superior to that of HA and other commercial humectant agents, probably because the EPS contains not only large amounts of glucuronic acid and *N*-acetyl glucosamine, the components of HA, but also fucose, which has a good moisturizing effect^31^. In addition, its complex structure reflected in its heterogeneous glycosyl composition and linkages may also contribute to the retention of bonded water in a spacious network^32^. The moisture-retention ability of SM1127 EPS is also superior to that of other reported EPSs^30^,^33^. The super moisture-retention ability of SM1127 EPS gives it promising potential to be used in the cosmetics field as a moisturizing ingredient and in the biomedicine field as wound dressing^34^.

Reactive oxygen species, such as hydroxyl and superoxide radicals, are highly related to human health. They may cause ageing, cancer, inflammation and other diseases^35^. Thus, it is important to develop synthetic or natural polymers with antioxidant activity to reduce the oxidative stress in the human body. More attention has been paid to natural polymers with antioxidant activity due to their higher safety. To date, most microbial polysaccharides with antioxidant activity have been reported from fungi^36^. Here, we found that SM1127 EPS has considerable antioxidant activity reflected in its good capacity for scavenging DPPH•, •OH and O_2_^–^•, which may be attributed to its functional groups such as -OH, -COOH, C = O and -O- in the structure. These groups can donate electrons to reduce the radicals to a more stable form or react with the free radicals to stop the radical chain reaction^37^. Its good free radical scavenging ability endows SM1127 EPS with potential in cosmetics as an anti-ageing ingredient, in functional foods as a natural agent^38^ and in the pharmaceutical industry as an antioxidant ingredient.

The EPSs from some polar microorganisms have been shown to have cryoprotective effects for microbial cells^17^,^18^,^19^. In this study, we investigated the low-temperature protective effect of SM1127 EPS on human dermal fibroblasts. Our results showed that SM1127 EPS could considerably increase the viability of human dermal fibroblasts at 4 °C. Human dermal fibroblasts are the main component of skin, which is essential for maintaining homeostasis and also plays an important role in the healing of cutaneous wounds^39^. Our results suggest that SM1127 EPS may protect human skin from cold injury.

In summary, the EPS of *Polaribacter* sp. SM1127, an Arctic marine bacterium, has heterogeneous glycosyl composition and glycosyl linkages. SM1127 EPS has good rheological properties and tolerance to a wide pH range and high salinity. Moreover, this EPS shows outstanding moisture-retention ability and good antioxidant activity. It also presents considerable protective effects on human dermal fibroblasts at low temperature. Safety evaluation shows that SM1127 EPS is nontoxic and nonirritating to skin. These properties indicate that SM1127 EPS has promising potential to be applied safely in the food, cosmetic, and biomedical fields.

## Methods

### Screening of EPS-producing strains

The 152 isolates used to screen EPS-producing strains were previously isolated from brown algae collected from the intertidal zone of Kings Bay in Ny-Ålesund, Svaldbard^40^. To screen EPS-producing strains, the isolates were cultured at 15 °C for 3 days on Congo red agar plates containing medium composed of 5 g/l peptone (Oxoid, England), 1 g/l yeast extract (Oxoid, England), 30 g/l glucose (Sinopharm, China), 0.8 g/l Congo red, 30 g/l sea salt (Sigma, America), 1.5 g/l agar and distilled water (pH 7.0)^41^. Strains forming smooth, humid and mucoid colonies on the plates were selected and further cultured at 15 °C and 200 rpm in basal marine medium for EPS production, which contained 30 g/l glucose, 10 g/l peptone, 5 g/l yeast extract and 30 g/l sea salt (pH 7.0)^42^. After a 5-day cultivation, bacterial cells were removed from the broth by centrifugation at 10,000 rpm and 4 °C for 10 min. EPSs were separated from the supernatant by addition of two volumes of chilled absolute ethanol^19^ and dissolved in deionized water. The EPS concentrations of the solutions were determined by using the phenol-sulfuric acid method with D-glucose as a standard^43^. Strain SM1127, with the highest EPS production, was chosen for further study.

### Phylogenetic identification of strain SM1127

Total genomic DNA was extracted from strain SM1127 using a bacterial genomic DNA extraction kit (BioTeke, China). The 16S rRNA gene was amplified from the genomic DNA by PCR using the 27F (5′-AGAGTTTGATCCTGGCTCAG-3′) and 1492R (5′-GGTTACCTTGTTACGACTT-3′) primers^44^. Then, the obtained 16S rRNA gene sequence was compared with those of other bacteria in GenBank using the BLAST program (http://blast.ncbi.nlm.nih.gov/Blast.cgi) to determine the phylogenetic affiliation of strain SM1127. A neighbor-joining phylogenetic tree based on the16S rRNA gene sequences of SM1127 and other related bacterial strains was constructed using MEGA 5.0 software^45^.

### EPS isolation and purification

Strain SM1127 was cultured in basal marine medium for EPS production at 15 °C and 200 rpm for 5 days. Bacterial cells were removed from the culture broth via a 10 min centrifugation at 10,000 rpm. EPSs were separated from the supernatant by the addition of two volumes of chilled absolute ethanol^19^ and lyophilized. The lyophilized precipitate was dissolved in deionized water (3%, w/v) and treated with 15 units/ml compound protease (Gold wheat, China) at 50 °C and 120 rpm for 5 h to exclude proteins. After further precipitation by cold absolute ethanol and lyophilization, the crude EPS of strain SM1127 was obtained.

The crude EPS was further purified by anion-exchange chromatography using a DEAE-Sepharose Fast Flow column (1.6 × 25 cm) with 0–0.7 M NaCl gradient as the eluant at a flow rate of 36 ml/h, followed by gel-filtration chromatography using a Sepharose 4B column (1.6 × 95 cm) with deionized water as the eluant at a flow rate of 12 ml/h. During these processes, the polysaccharide content in the fractions was monitored quantitatively by using the phenol-sulfuric acid method^43^, and protein content was monitored by UV and visible (UV-V is) absorption spectra on a Jasco V-550 spectrophotometer. The purified EPS was dialyzed in deionized water and lyophilized.

The purity of the EPS was analyzed on a Shimadzu analytical HPLC system with a Shimadzu autoinjector using a Waters Ultrahydrogel linear column (7.8 × 300 mm) at 40 °C and detected by a Shimadzu refractive index detector.

### Analysis of the glycosyl composition, glycosyl linkage and molecular mass of the EPS from strain SM1127

Glycosyl composition analysis of the EPS from strain SM1127 was carried out as described by York *et al.*^46^ and Merkle & Poppe^47^. The purified EPS (500 μg) was placed in a separate tube with 20 μg of inositol as the internal standard. Trifluoroacetic acid (2 M, 400 μl) was added, and hydrolysis was carried out at 120 °C for 1 h. Then, the sample was lyophilized. Methyl glycosides were prepared from the dry sample by methanolysis in 1 M HCl at 80 °C for 16 h, followed by re-*N*-acetylation with pyridine and acetic anhydride in methanol. The sample was then per-*O*-trimethylsilylated with Tri-Sil (Pierce, America) at 80 °C for 0.5 h. Combined gas chromatography/mass spectrometry (GC/MS) analysis of the per-*O*-trimethylsilyl derivatives was performed on an Agilent 7890A GC interfaced to a 5975C mass selective detector (MSD) using a Supelco EC-1 fused-silica capillary column (0.25 mm × 30 m).

For glycosyl linkage analysis, the purified EPS were permethylated, depolymerized, reduced, and acetylated. The resultant partially methylated alditol acetates (PMAAs) were analyzed with GC/MS^46^. Firstly, the purified EPS (2 mg) was suspended in 200 μl dimethyl sulfoxide (DMSO) and purged with a nitrogen stream. The sample was permethylated by the addition of 1 ml Hakomori base while purging with a nitrogen stream and mixed for 7 h. CH_3_I was then added and mixed overnight. The sample was passed through a C18 SEP-PAK and dried down. After reduction with lithium borodeuteride in tetrahydrofuran, the sample was neutralized, evaporated and re-permethylated with NaOH and CH_3_I in dry DMSO^48^. Then, the sample was subjected to NaOH base for 15 min and supplemented with CH_3_I standing for 40 min. The permethylated material was hydrolyzed with 2 M trifluoroacetic acid, reduced with NaBD_4_, and acetylated with acetic anhydride. The resulting PMAAs were analyzed on an Agilent 7890A GC interfaced to a 5975C MSD (electron impact ionization mode), and the separation was performed on a 30-m Supelco 2380 bonded-phase fused-silica capillary column.

The molecular mass (MW) of the EPS was estimated by size-exclusion chromatography with a TSK Gel G5000PW column (7.5 × 300 mm, Tosoh Biosciences) equilibrated with 50 mM ammonium acetate buffer (pH 5.5). After calibration of the column with dextran MW standards (1189, 759, 511 and 167 kDa) and glucose, 50 μl EPS solution (2 mg/ml) was loaded onto the column and was eluted with 50 mM ammonium acetate buffer (pH 5.5) at 1 ml/min. An ELS detector (Agilent, America) was used for post-column detection of soluble components eluted from the column, and data were collected and processed by Agilent ChemStation software.

### Property analysis of crude EPS

Different concentrations [0.5%, 0.8%, 1.0%, 1.2%, 1.5% and 2.0% (w/v)] of the crude EPS solution were prepared, and the viscosity of these samples under different shear rates (1, 2, 10, 15, 20, 40 and 60 rpm) was measured by using a Brookfield viscometer (model LVDVII + P; Brookfield Engineering Laboratories, America) with spindle S16 at 25 °C. To investigate the pH stability of the EPS, 10 M HCl and 10 M NaOH were used to adjust the pH of the 1.5% (w/v) EPS solutions. To investigate the salt tolerance of the EPS, 1.5% (w/v) EPS solutions of different electrolyte concentrations were made with NaCl (200 g/l) and CaCl_2_ (200 g/l), and the viscosity of these EPS solutions was measured using a Brookfield viscometer with spindle S16 at a shear rate of 20 rpm.

### Moisture-absorption and retention ability of crude EPS

The moisture-absorption and retention ability of the crude EPS and control samples were studied using the method reported by Zhao *et al.*^30^. Prior to the moisture-absorption test, the crude EPS from strain SM1127 and the control samples, including HA, chitosan, sodium alginate and glycerol, were pulverized to 80 mesh and dried over P_2_O_5_ *in vacuo* for 24 h. Then, 500 mg dried samples were put in a saturated K_2_CO_3_ chamber [43% relative humidity (RH)] and a saturated (NH_4_)_2_SO_4_ chamber (81% RH) at 25 °C, respectively. The water-absorption ability (*R*_*a*_) was determined by the percentage of weight increase of dry sample:


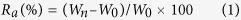


where *W*_*0*_ and *W*_*n*_ are the weight of a sample before and after being put in the chamber. Samples were consecutively tested at different time points for 72 h.

In the moisture-retention test, the dried EPS and the control samples were kept in 43% RH and weighed at different time points for 72 h. The moisture-retention ability (*R*_*h*_) was determined by the percentage of residual water of wet samples:


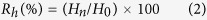


where *H*_*0*_ and *H*_*n*_ are the weight of water in the sample before and after being put in the desiccator with allochroic silica gel at 25 °C.

### Antioxidant activity of crude EPS

The free radical-scavenging activities for 1,1-diphenyl-2-picryl-hydrazyl radical (DPPH•), hydroxyl radical (•OH) and superoxide anion (O_2_^–^•) were assessed as indicators of the antioxidant activity of the crude EPS from SM1127. HA, which is able to scavenge radicals^49^, was used as a positive control. According to the method reported by Braca *et al.*^50^, 1 ml samples with different concentrations (0.1, 0.25, 0.5, 1.0, 2.0, 3.0, 5.0, 7.5 and 10.0 mg/ml) were mixed with 2 ml ethanol solution of DPPH (100 μM). After being in the dark for 40 min at 25 °C, the absorbance was recorded at 525 nm. The scavenging activity for •OH of the samples was determined by the FeSO_4_-salicylic acid method^51^. In the assay, 1 ml FeSO_4_ solution (9 mM) and 1 ml ethanol solution of salicylic acid (9 mM) were mixed with 1 ml sample. Then, 1 ml H_2_O_2_ solution (8.8 mM) was added to the mixtures to start the reaction. After a 30-min incubation at 37 °C, the absorbance was recorded at 510 nm. The scavenging activity for O_2_^–^• of the samples was studied by the pyrogallol autoxidation method^52^. In the experiment, 1 ml sample and 0.4 ml HCl solution (10 mM) of pyrogallol (25 mM) were mixed with 4.5 ml Tris-HCl buffer (50 mM, pH 8.2). After a 5-min incubation at 25 °C, 1 ml HCl (8 mM) was added to terminate the reaction and then the absorbance at 320 nm was recorded.

The free radical-scavenging activity (*D*) was calculated as follows:


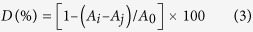


Where *A*_*i*_ *is* the absorbance of the sample, *A*_*j*_ is the background absorbance of the sample and *A*_*0*_ is the absorbance of the blank control.

### Low-temperature protective effect of crude EPS on human dermal fibroblasts

Human dermal fibroblasts were grown in Roswell Park Memorial Institute (RPMI) 1640 medium with 10% fetal bovine serum (FBS) (Invitrogen, America) at 37 °C and 5% CO_2_ for 2 days and maintained to 80–90% confluence. Different concentrations (0, 100, 200 and 500 μg/ml) of the crude EPS from SM1127 were added into the culture, which was then incubated at 4 °C. After a 20-h incubation, the cells were stained with fluorescein isothiocyanate (FITC)-labeled Annexin V and simultaneously with propidium iodide (PI). Then, they were measured using a bivariate flow cytometer (ImageStream^x^ mk 65; Amnis, Germany).

### Safety assessment of crude EPS

An acute toxicity test and a skin irritation test were performed for safety assessment of the crude EPS from strain SM1127. Acute oral toxicity of the crude EPS was measured according to the Good Laboratory Practice Standards manual and Organization for Economic Cooperation and Development (OECD) Guidelines for Acute Toxicity of Chemicals no. 420^53^. KM female mice (8 weeks old, weight between 19 and 21 g) were obtained from Experimental Animal Center of Shandong University (Jinan, China). The control and the treated groups each contained five animals. A dose of 5000 mg/kg of the EPS for oral administration was used for the test. The control group received deionized water in the same dose as the treated group. The development of abnormal behavior, changes in skin color or body weight and any other toxicological effects were observed individually for 14 days. Animals were weighed on the 0, 7^th^ and 14^th^ days. At the end of the experiment, all animals were euthanized by carbon dioxide asphyxiation, their organs including heart, kidney, liver and spleen were processed in wax blocks and serial transverse sections were prepared, which were then stained with hematoxylin-eosin (HE) and observed by light microscopy (Axio Imager. A2; Zeiss, Germany). The histopathological results of all treated animals were compared with those of the control animals.

Skin irritation tests of the crude EPS were performed according to the Good Laboratory Practice Standards manual and OECD Guidelines for Acute Dermal Irritation of Chemicals no. 404^54^. Ten Japan white rabbits (1.6–1.8 kg, half male and half female) were obtained from Experimental Animal Center of Shandong University (Jinan, China). Approximately 24 h before the test, the hair (3 × 3 cm) of each animal was removed by closely clipping the dorsal area of the trunk on both sides. One dorsal area (the treated) was treated with 0.5 ml EPS-deionized water solution (125 mg/ml), covered with gauze and cellophane and fixed with bandages. In the meantime, the opposite dorsal area (the control) was treated with 0.5 ml deionized water. After 4 h, the EPS on the treated skin was removed with deionized water, then any signs of erythema or edema were recorded, and scores were given after 1, 24, 48 and 72 h according to Supplementary Table S1 online. The primary cutaneous irritation index (PII) was the average score, that is, total scores of erythema or edema were divided by the number of animals. For cumulative cutaneous irritation testing, the above once daily treatment was continued for 14 days^55^. After removing the EPS daily, any signs of erythema or edema were recorded, and scores were given 1 h later. The cumulative cutaneous irritation index (CII) was the average score, that is, total scores of erythema or edema were divided by the number of animals and testing days. Subsequently, the irritation intensity of the EPS was evaluated according to Supplementary Table S2 online.

## Additional Information

**How to cite this article**: Sun, M.-L. *et al.* Characterization and Biotechnological Potential Analysis of a New Exopolysaccharide from the Arctic Marine Bacterium *Polaribacter* sp. SM1127. *Sci. Rep.* **5**, 18435; doi: 10.1038/srep18435 (2015).

## Supplementary Material

Supplementary Information

## Figures and Tables

**Table 1:** Glycosyl composition of the EPS from strain SM1127.

Glycosyl residue	Amt^a^ (mol %)
Rhamnose (Rha)	0.8
Fucose (Fuc)	7.4
Glucuronic Acid (GlcA)	21.4
Mannose (Man)	23.4
Galactose (Gal)	17.3
Glucose (Glc)	1.6
*N*-Acetylglucosamine (GlcNAc)	28.0

^a^Amounts are expressed as the mole percent of total carbohydrates.

**Table 2:** Glycosyl linkage of the EPS from strain SM1127.

Glycosyl residue	Peak Area %
Terminal Fucopyranosyl residue (t-Fuc)	9.2
Terminal Mannopyranosyl residue (t-Man)	5.6
Terminal Glucopyranosyl residue (t-Glc)	1.0
4 linked Fucopyranosyl residue (4-Fuc)	7.9
Terminal Galactopyranosyl residue (t-Gal)	11.5
2 linked Rhamnopyranosyl residue (2-Rha)	0.2
3 linked Mannopyranosyl residue (3-Man)	1.8
3 linked Galactopyranosyl residue (3-Gal)	7.9
2 linked Galactopyranosyl residue (2-Gal)	13.1
4 linked Glucopyranosyl residue (4-Glc)	10.2
4 linked Glucuronopyranosyl residue (4-GlcA)	13.2
2,3 linked Mannopyranosyl residue (2,3-Man)	8.5
4,6 linked Manopyranosyl residue (4,6-Man)	0.6
Terminal *N*-Acetyl glucosamine residue (t-GlcNAc)	2.9
4 linked *N*-Acetyl glucosamine residue (4-GlcNAc)	6.5
