# Supplementary material for: Characterization and Biotechnological Potential Analysis of a New Exopolysaccharide from the Arctic Marine Bacterium Polaribacter sp. SM1127
Source: Sci Rep. 2015 Dec 21;5:18435. doi: 10.1038/srep18435 (PMC4685270; doi:10.1038/srep18435)
Supplement: Supplementary Information [file srep18435-s1.doc]

**Characterization and Biotechnological Potential Analysis of a New Exopolysaccharide from** **the Arctic Marine Bacterium *Polaribacter* sp. SM1127**

Mei-Ling Sun^1,2^, Fang Zhao^1,2^, Mei Shi^1,2^, Xi-Ying Zhang^1,2^, Bai-Cheng Zhou^2^, Yu-Zhong Zhang^1,2^, Xiu-Lan Chen^1,2,*^

^1^State Key Laboratory of Microbial Technology, ^2^Marine Biotechnology Research Center, Shandong University, Jinan 250100, China

**Supplementary Data**


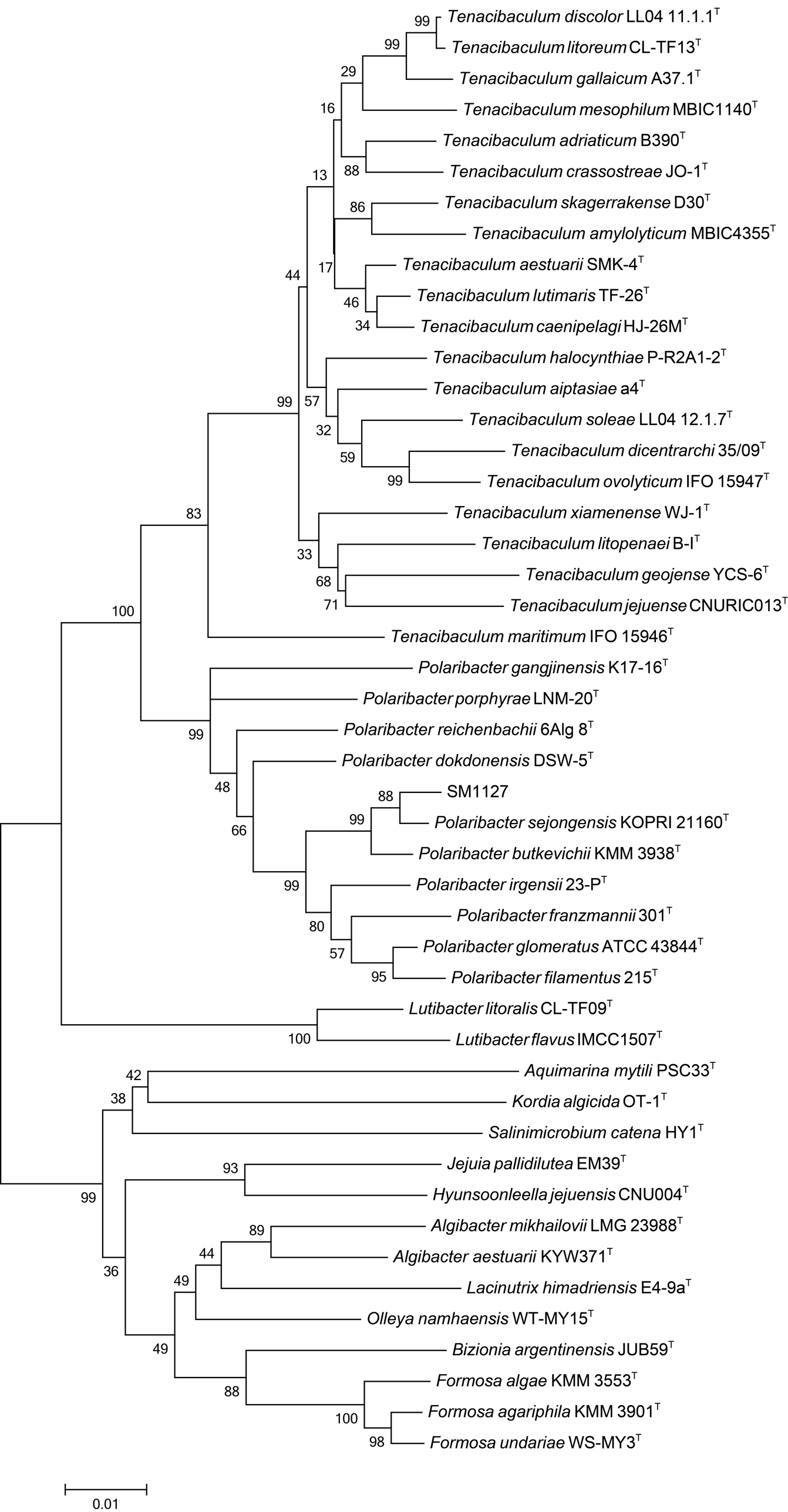


**Figure S1. The neighbour-joining phylogenetic tree based on 16S rRNA gene sequences showing the phylogenetic position of strain SM1127.**


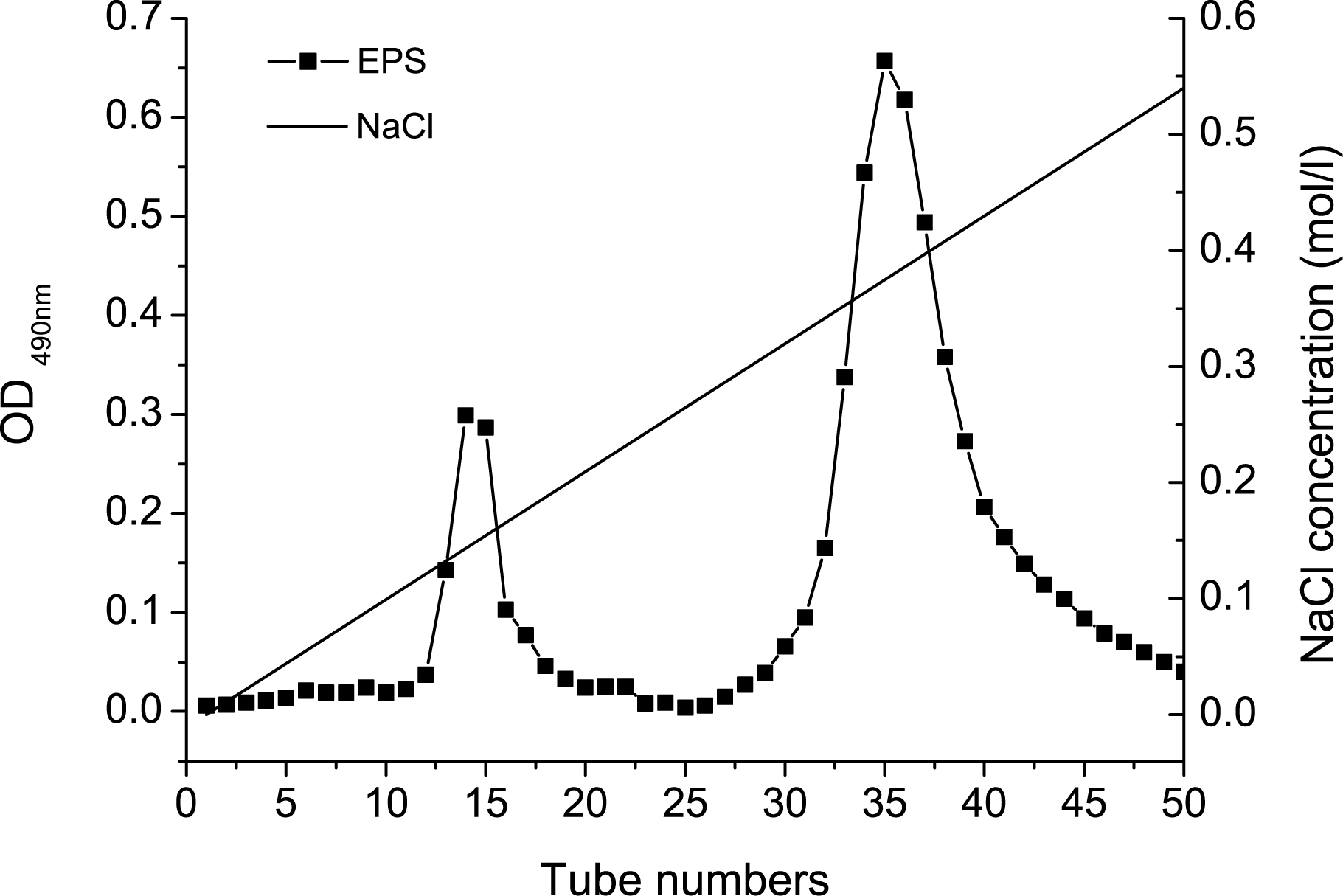


**Figure S2. Anion-exchange chromatography (DEAE-Sepharose Fast Flow) of the EPS from strain SM1127.** The EPS was eluted with a linear gradient of 0 to 0.7 M NaCl aqueous solution at a flow rate of 36 ml/h.


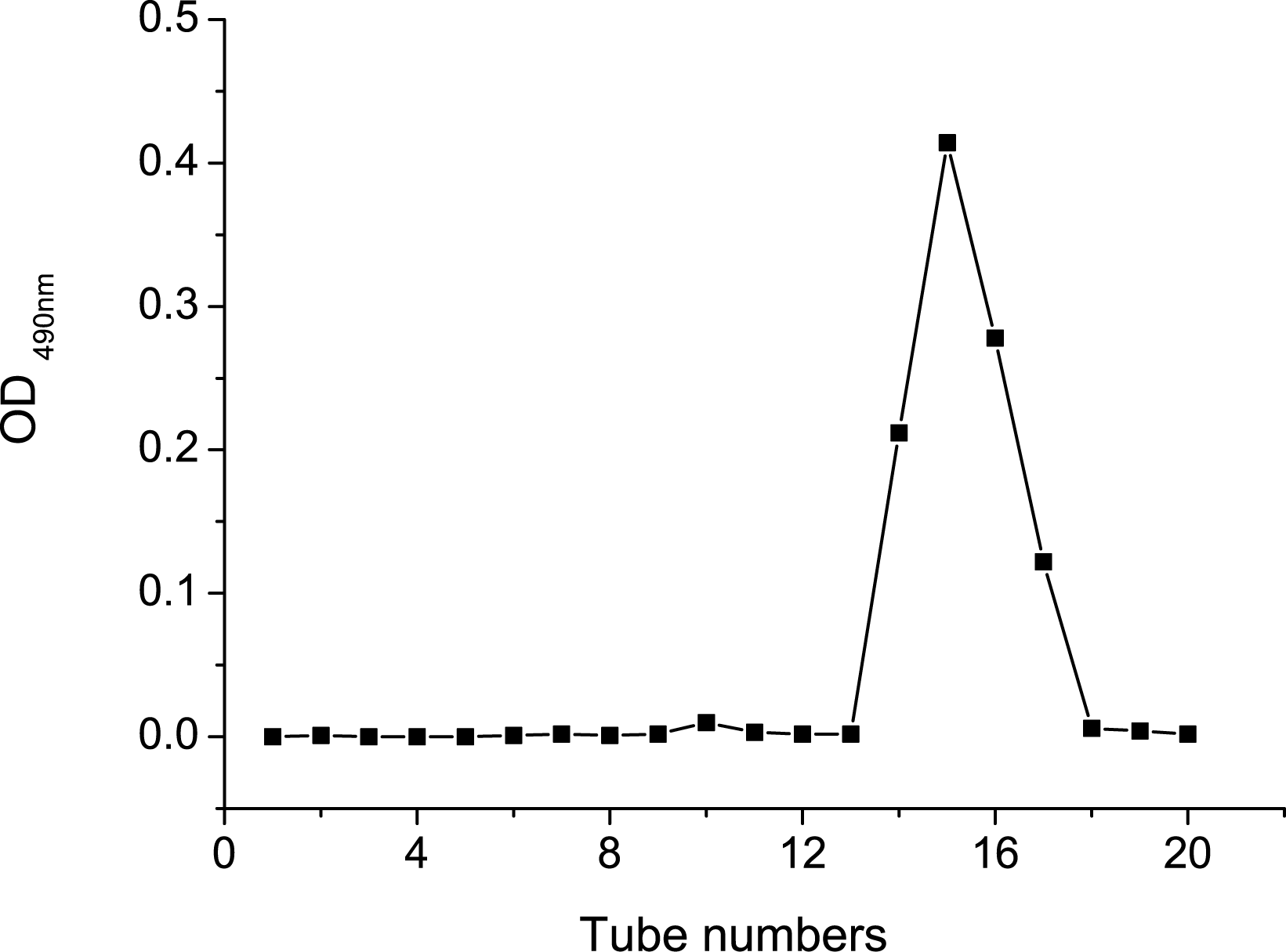


**Figure S3. Gel-filtration chromatography (Sepharose 4B) of the EPS from strain SM1127.** The EPS was eluted with deionized water at a flow rate of 12 ml/h.


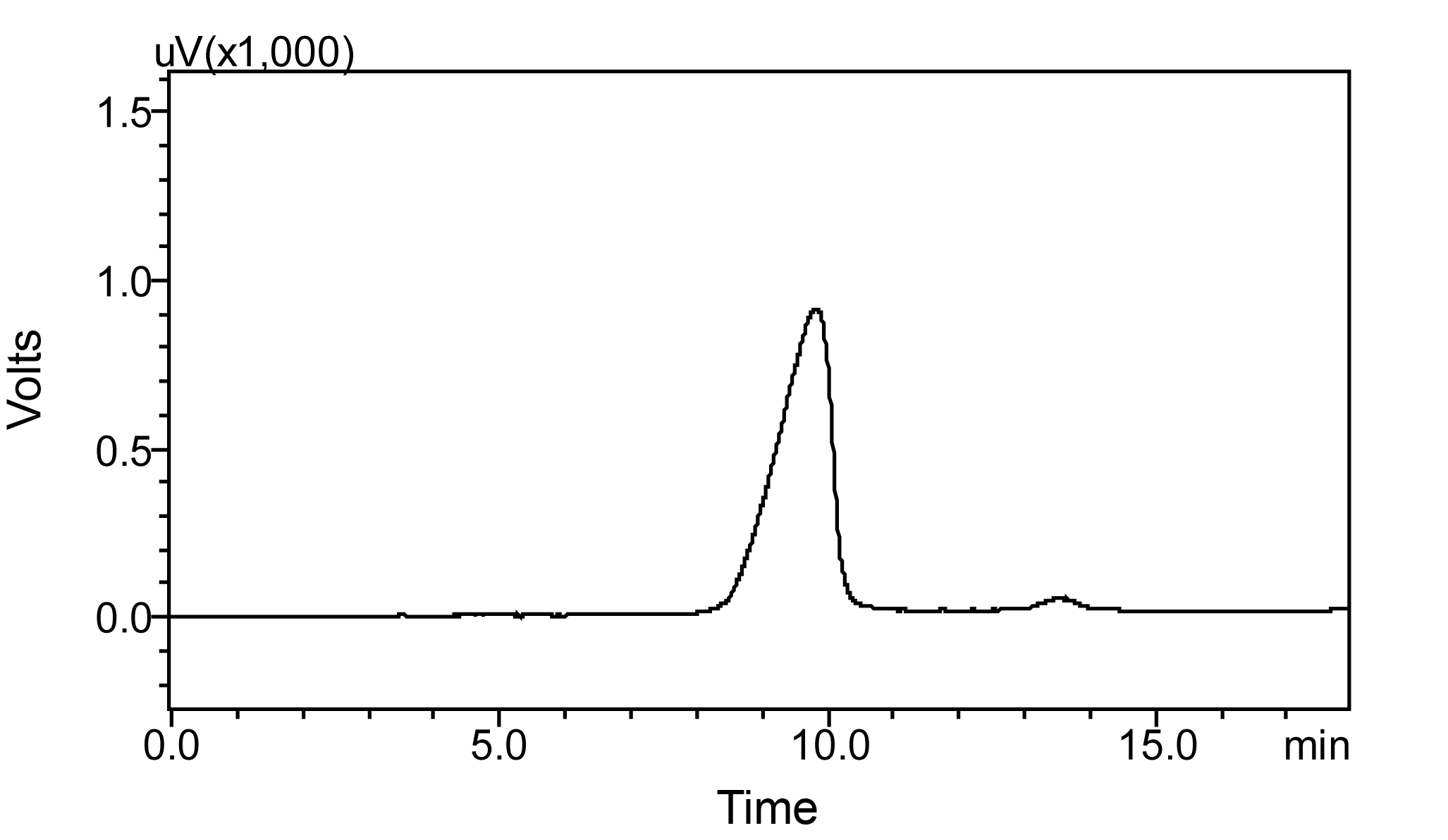


**Figure S4. Homogeneity analysis of the purified EPS from strain SM1127 by a Shimadzu analytical HPLC system.** The EPS was dissolved in deionized water, prepared as 0.5% (w/v) aqueous solution and 20 μl of the sample solution was injected in each run process for 30 min at a flow rate of 0.5 ml/min at 40°C. The sample was monitored by a Shimadzu refractive index detector.


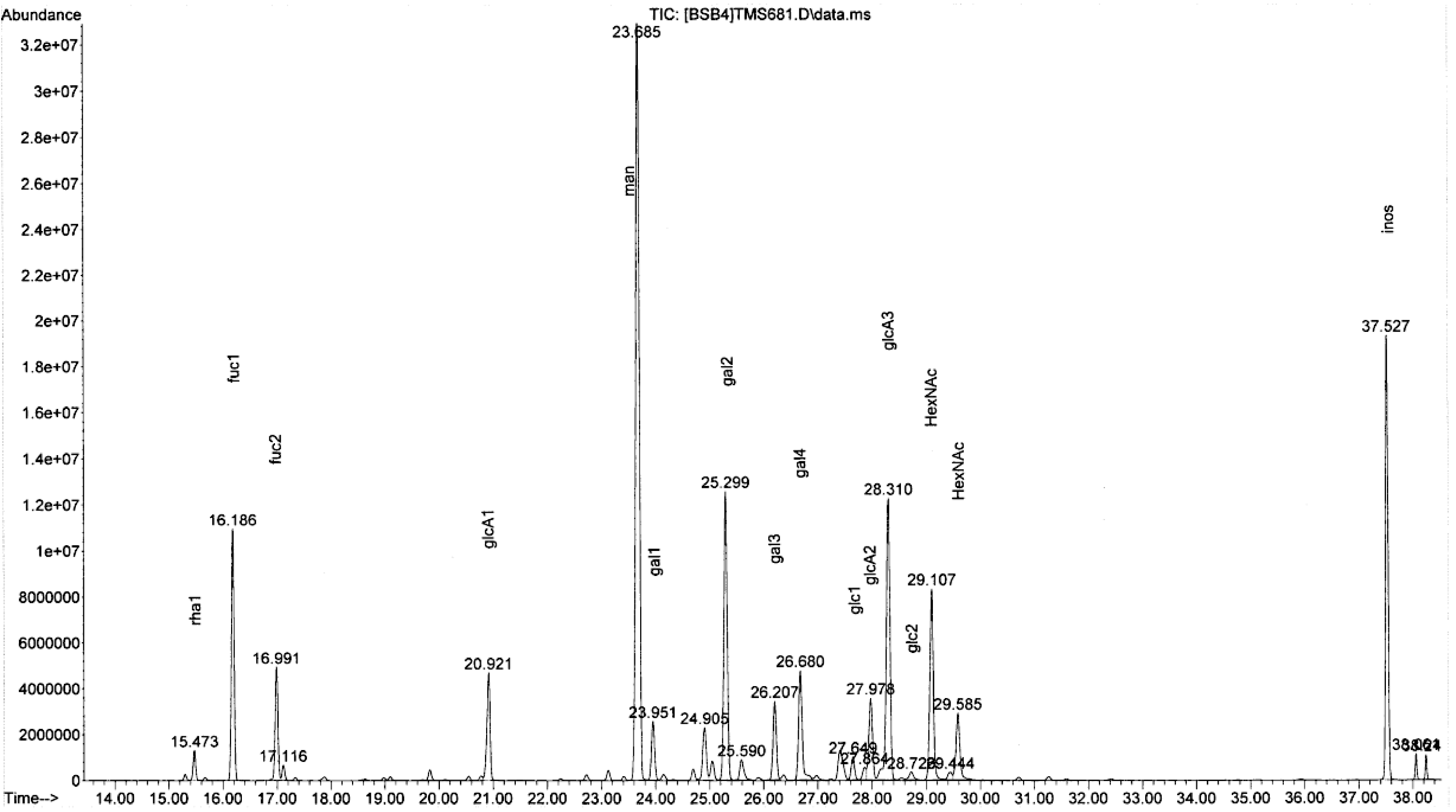


**Figure S5. GC/MS spectrum of glycosyl composition analysis of the EPS from strain SM1127 on a Agilent 7890A GC interfaced to a 5975C MSD, using a Supelco EC-1 fused-silica capillary column (0.25 mm × 30 m).**


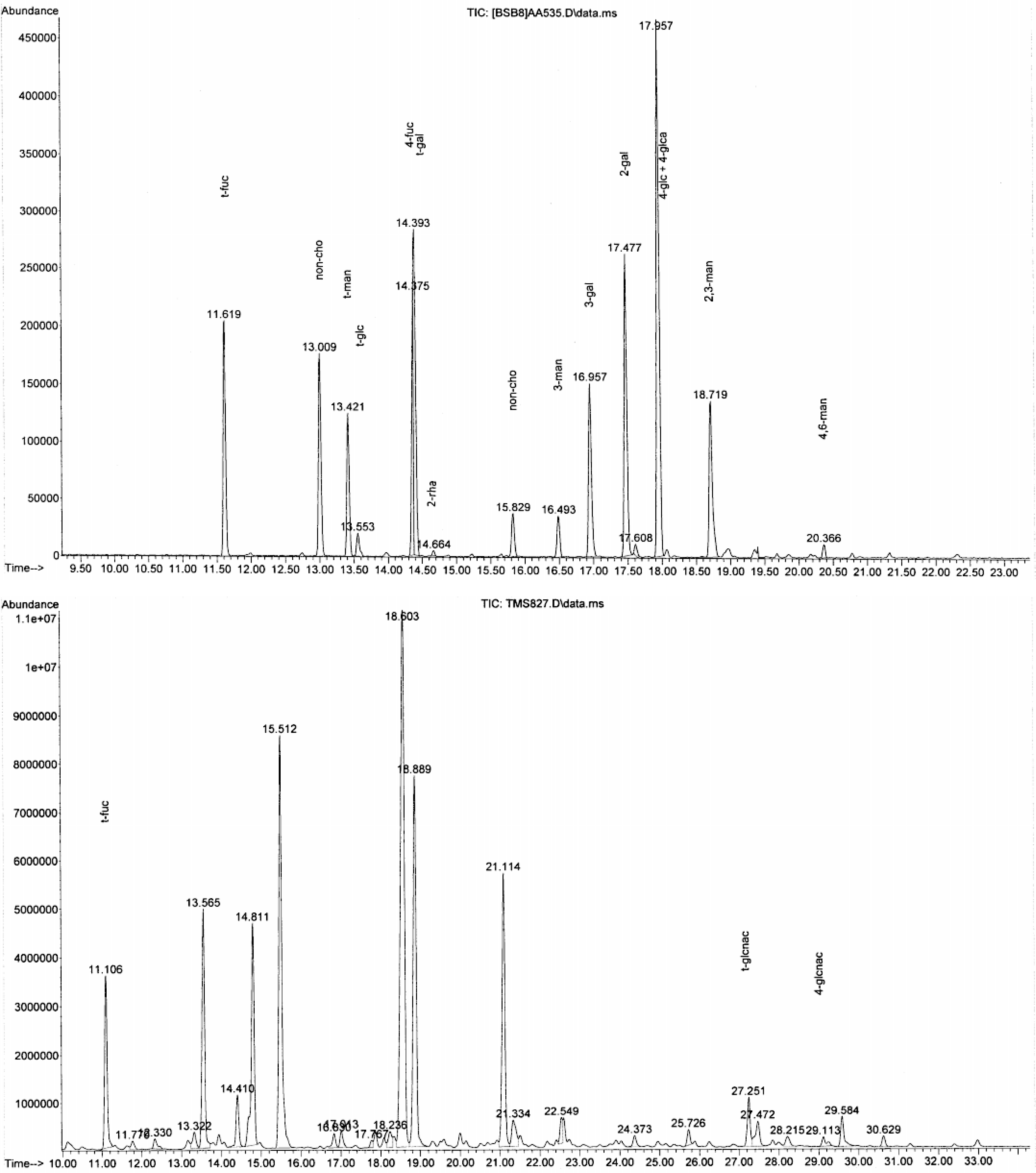


**Figure S6. GC/MS spectrum of glycosyl linkage analysis of the EPS from strain SM1127 on an Agilent 7890A GC interfaced to 5975C MSD (electron impact ionization mode), using a 30-m Supelco 2380 bonded-phase fused-silica capillary column.**


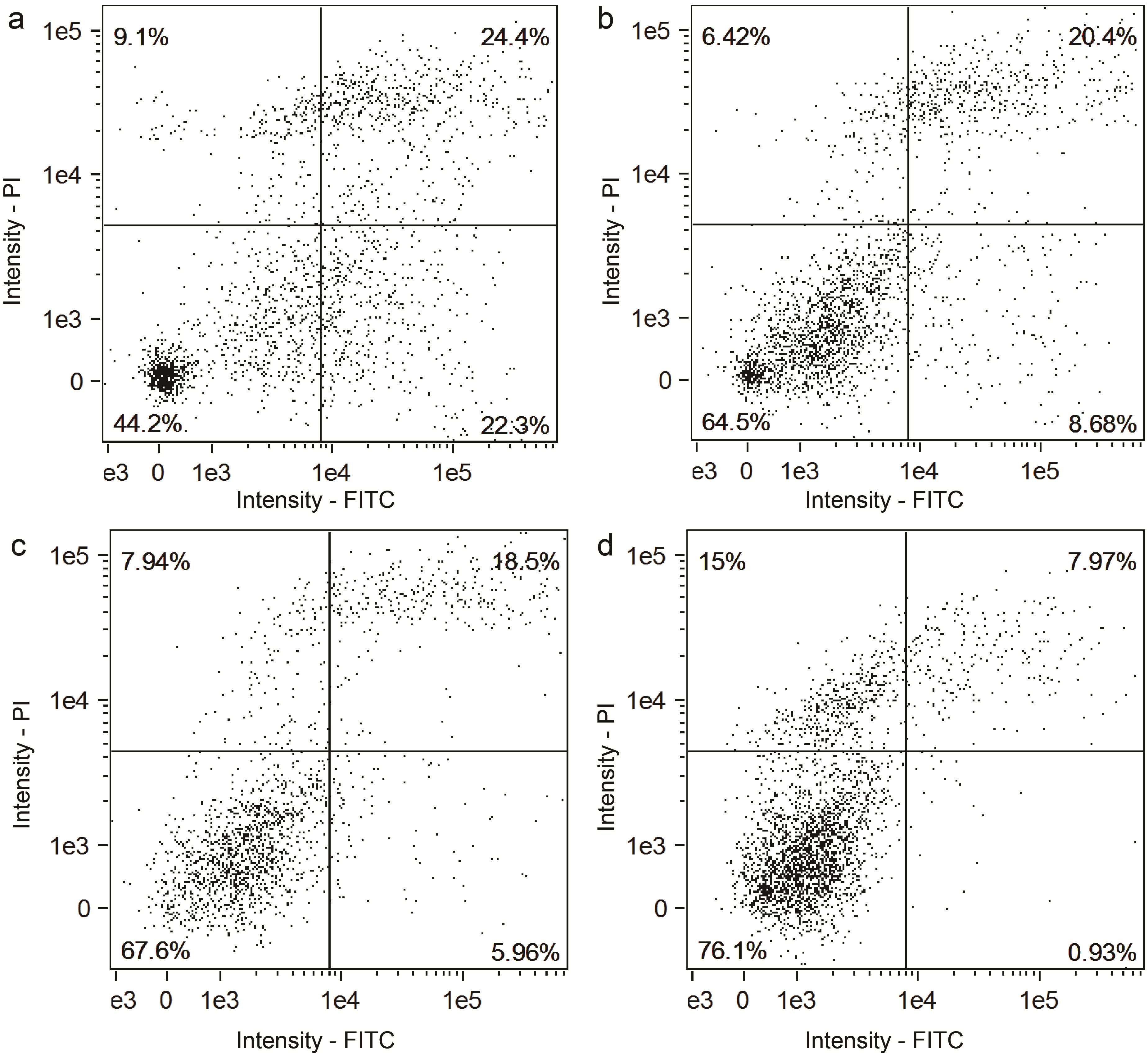


**Figure S7. Analysis of the protective effect of the SM1127 EPS on human dermal fibroblasts at 4°C by flow cytometer.** EPS concentration was (A) 0 µg/ml, (B) 100 µg/ml, (C) 200 µg/ml and (D) 500 µg/ml. The lower left quadrant showed the viable cells, which excluded PI and was negative for Annexin V-FITC binding. The upper right quadrant showed the necrotic cells, positive for Annexin V-FITC binding and for PI uptake.

**Table S1. Grading standard for skin irritation according to OECD guidelines^54^**

| Irritation response | Score |
| --- | --- |
| Erythema |  |
| No | 0 |
| Very slight (barely perceptible) | 1 |
| Well defined | 2 |
| Moderate to severe | 3 |
| Severe (beef redness) to eschar formation preventing grading of erythema | 4 |
| Edema |  |
| No | 0 |
| Very slight (barely perceptible) | 1 |
| Slight (edges of area well defined by definite raising) | 2 |
| Moderate (raised approximately 1 mm) | 3 |
| Severe (raised more than 1 mm and extending beyond area of exposure) | 4 |
| Highest total value | 8 |

**Table S2. Evaluation standard for skin irritation intensity according to OECD guidelines^54^**

| Score | Evaluation |
| --- | --- |
| 0–0.49 | No irritation |
| 0.5–2.99 | Slight |
| 3.0–5.99 | Moderate |
| 6.0–8.00 | Severe |
